# Seed nano-priming with Zinc Oxide nanoparticles in rice mitigates drought and enhances agronomic profile

**DOI:** 10.1371/journal.pone.0264967

**Published:** 2022-03-24

**Authors:** Muhammad Waqas Mazhar, Muhammad Ishtiaq, Iqbal Hussain, Abida Parveen, Khizar Hayat Bhatti, Muhammad Azeem, Sumaira Thind, Muhammad Ajaib, Mehwish Maqbool, Tauqeer Sardar, Khursheed Muzammil, Nazim Nasir

**Affiliations:** 1 Department of Botany, Mirpur University of Science & Technology (MUST), Mirpur, AJK, Pakistan; 2 Department of Botany, Government College University Faisalabad, Faisalabad, Pakistan; 3 Department of Botany, University of Gujrat, Gujrat, Pakistan; 4 Department of Biology, College of Science, University of Bahrain, Zallaq, Bahrain; 5 Department of Public Health, College of Applied Medical Sciences, Khamis Mushait Campus, King Khalid University, Abha, Saudi Arabia; 6 Department of Basic Medical Sciences, College of Applied Medical Sciences, Khamis Mushait Campus, King Khalid University, Abha, Saudi Arabia; Anhui Agricultural University, CHINA

## Abstract

All cereal crops, particularly rice are perpetually affected due to drastic climatic changes which triggers different stressors resulting in food shortage scenarios across the globe. In modern era, application of nanotechnology holds the pledge in combating the climate change mediated environmental stressors through nanomaterials such as pesticides, nano-biosensors, nano-clays and nano-seed priming technologies. Current study is a part of experiment conducted to comprehend the behaviour of rice plants raised from Zinc Oxide nanoparticles (ZnONPs) primed seeds under the water shortage environment. The seed priming treatment concentrations included 0, 5, 10, 15, 25 and 50 ppm. In the experimental results an increase in plant height, total chlorophyll contents, plant fresh and dry weights was obtained by use of seed priming with ZnONPs. The study results proved that seed priming with 25ppm of ZnONPs increased seed and straw yield with value of 85.333 and 123.333, respectively under water deficit environment. The analysis depicted that 25 ppm has been found more suitable for increasing the 1000 paddy weight of rice plants under both well irrigated and water shortage conditions. Seed priming with ZnONPs results in 53% reduction in MDA contents of water stressed rice plants Drought stress leads to reduction in plant height by 31%, plant fresh weight by 22% and plant dry weight by 28%. Seed priming treatments imparted in current study show significance increase in plant biomass. Priming with ZnONPs further enhances the levels of proline amino acid facilitating the plant to combat water shortage stress. A further elevation in activities of SOD, CAT and POD takes place in rice plants raised from ZnONPs primed seeds by 11%, 13% and 38%, respectively. An elevation in activities of antioxidant enzymes was found and the levels of oxidative stress indicators decreased upon seed priming with ZnONPs. Furthermore the yield characteristics such as panicle length, number of tillers, paddy yield and straw yield of the rice plants raised through ZnONPs primed seeds enhanced. The ZnONPs at concentration of 25 ppm proved optimum in alleviating drought induced damages. It can be inferred that seed pre conditioning with ZnONPs is helpful in increasing yield attributes under the water shortage environment.

## 1-Introduction

Climate change is posing extreme environmental challenges to wild plants and crops such as frequent droughts temperature increase, rise of CO_2_ conc. and fluctuating weather patterns worldwide. The Intergovernmental Panel on Climate Change (IPCC) has warned that CO_2_ concentration will reach up to 550 ppm by year 2050 and there will be elevation in temperature of globe in the range of 2-to-5°C [1]. Climate change (CC) is going to threaten the agricultural countries of Asia and Africa bringing frequent water shortages and erratic rains impact severe threat of food security.

Drought affects more than one third percent of croplands across the globe causing food threat to people [2]. The previous research work depicted that water shortage leads to average yield losses up to 50% worldwide [3]. It is reported that water deficit scenario has led to drop in plant metabolism, stomata conductance and gaseous exchange leading towards poor agronomic traits [4]. Usually, water shortage environment leads to production of Reactive Oxygen Species (ROS) which may act as mutagen as well as destructive for plant metabolism as it leads to enzymatic denaturation [5]. ROS leads to lipid peroxidation of biological membranes affecting transport processes across the membranes [6]. Under water deficit environment plant increase activities of antioxidant enzymes such as Superoxide Dismutase (SOD), Peroxidase (POD), and Catalase (CAT) [2]. Plants accumulate the sugar osmolytes such as proline and vitamins in their cytosol as internal defence mechanism to ROS mediated oxidative stresses [7]. The seed priming treatments and foliar spray with osmolytes and minerals leads to check in ROS production and stress mitigation [8].

Rice (*Oryza sativa* L.) is a cereal providing food security to more than half of the world population [9]. The rice is a source of caloric intakes for more than 520 million people living in Asian countries [10]. In Pakistan, rice is the second major crop after wheat and it is cultivated on 2.531 million hectares producing an average of 5.5 million tonnes of paddy grains per annum [11]. About 90 percent of the worldwide rice production is from Asian countries and the Asian countries such as Pakistan are the most vulnerable to climate change mediated weather patterns [12].

Since the last decade nanotechnology has emerged as a fascinating field in agronomy and crop industry [13]. The research momentum has changed dramatically toward green synthesis of nanoparticles and their application in alleviating abiotic and biotic constraints [14]. Nanoparticles are serving as magic bullets in transforming agricultural world with novel set of nanomaterial which can serve as potential candidates in stress mitigation and in increasing food production [15]. The potential of nanoparticles in alleviating drought has been reported by various researchers [16–18].

The mechanism of Zinc functioning is presented in many previous papers and it is inevitable for many life functions of the plants. It is stated that Zn is an essential nutrient for normal homeostasis of plants as it is actively involved in boosting metabolism of proteins, biosynthesis of hormones and cofactor for enzymes [19]. For normal homeostasis plants require zinc at concentration of 27-150mg per Kg biomass [20]. Zinc improves activities of SOD by acting as activator and it improves plant water relationship [21]. Zinc is helpful in alleviating ROS induced damages and improves nutrient profiles of cereals [22].

Seed pre-conditioning is an efficient modulation effect against oxidative stress damages faced by the plants [23]. Seed priming with biologically active compounds is an efficient tool to combat oxidative stress mediated damages [24]. To the best of our knowledge little work has been reported on seed priming with nanoparticles and their role in combating stresses especially in perspective of climate change. The hypothesis is cultivation of ZnONPs primed seeds might alleviate water deficit mediated yield losses in rice and current study is aimed to explore the potential of ZnONPs to be used as seed priming agent in rice specifically in Pakistan where almost all rice croplands are Zinc deficient and subsequently it has drastic effects in mitigating drought stress hazards and improving yield.

## 2-Materials and methods

### 2.1-Experimental layout

Current study was conducted in field experimental trial plots at Department of Botany Mirpur University of Science and Technology (MUST),of District Bhimber (33°9′0.72″N 73°44′41.53″E), Azad Jammu and Kashmir (AJK), Pakistan. Experimental conditions were having mean day and night temperature of ca. 35°C and 28°C, respectively. The rice plants were grown under experimental conditions of 12 hours of photoperiod exposure. The seeds of rice variety IRRI-6 were obtained from National Agricultural Research Centre (NARC) Islamabad. In the experiment seeds (150 seeds per 100mL) were dipped thrice in 10% solution of H_2_O_2_ and immersed in 1% Sodium hypochlorite soln. for period of one hour. After this, seeds were rinsed in double distilled water (ddw) three to five times to remove excessive soln. immersion. In the experimental, for control trial sterilization step with Sodium hypochlorite was not performed while in second control trial 2% Sodium hypochlorite soln. was added to remove the excessive hypochlorite coating materials [25]. For soil structural and physiochemical analysis, about 10 Kg of upper 10-20cm soil was taken from farmland area of Bhimber AJK and the soil was air dried and sieved (2mm sieve). The soil characteristics were examined and found to have pH of 7.45, EC of 1.99 dsM^-1^. The total organic matter was around 1.98%. The soil samples were found to have ratio of sand silt and clay with respect to 36:41:23%. The soil was put in plastic pots having diameter of 26cm. Soil was supplied with basal dressing treatment of Nitrogen-Phosphorus Potassium (NPK) at the rate of 70kg of Nitrogen, 40kg of P_2_O_5_ and 25kg of K_2_O per hectare in the form of Urea, TSP and K_2_SO_4_, respectively (Table 1). Half of the nitrogen was given at the time of seed sowing and remaining half of the nitrogen was provided at the time of panicle initiation and/or emergence. Rice seeds were given seed priming treatments and were cultivated. The pot experiment was piloted in a randomized layout with three replicates per treatment. Nine seeds were planted per pot and after periodic thinning their number reduced to 5 plants per pot. Three plants were selected as treatment replicates from each pot [26, 27].

**Table 1:** Characteristics of experimental soil used for rice production.

Soil Character	Recorded Value
Soil Type	Loam
EC	1.99 dsM^-1^
pH	7.46
Organic Matter	1.98%
Sand	36
Silt	41
Clay	23

### 2.2-Drought treatment

The water holding capacity (WHC) of well irrigated pots was maintained at 70% with double distilled water and the pots having drought treatment experienced water holding capacity maintenance at 35% with distilled water following protocols of Adrees *et al*., [26]. continuously from the time of sowing up to harvesting.

### 2.3-Treatment application

ZnONPs were purchased from Alpha Genomics Plot 4-C, Main PWD Rd, Islamabad, Punjab Pakistan. The characterization of ZnONPs data suggested that the particles size was in range of 20-30nm with 98% purity level. The density of ZnONPs was ca. 5.2 km^-3^. For seed priming treatment different concentration of ZnONPs were prepared which include 0ppm as control treatment, while other trials were 5, 10, 15, 25 and 50 ppm applied on experimental units. Initially a little volumetric quantity of ZnONPs was put in de-ionized water. The mixture was ultrasonicated for 30 minutes to make uniform dispersions and subsequently the desired concentrations of ZnONPs were raised, as prepared as per adopted standard protocols. The control seeds were soaked in de-ionized water while rest of the seeds were dipped in their respective concentration range for 24hours under dark provided with continuous aeration treatment during priming [27].

### 2.4-Biomass production

Plant fresh and dry weights (Biomass production) was noted using a manual electronic and digital balance. After observing the fresh weights (FWs) of replicates the samples were subjected to high temperature (70°C) with the help of an oven for a time duration of 48 hours and then their dry biomass (DWs) was measured. A scale (meter rod) was used to measure the plant length in centimetres or in mm, where required. The samples were labelled carefully with respect to each treatment applied and their length was recorded in field notebook [2].

### 2.5-Analysis of Total Chlorophyll Content (TCC)

By using protocol devised by Arnon [28], the total chlorophyll contents of each experimental trial’s plants were assayed. The chlorophyll concentration in plant leaves was determined after 25 days of germination in plastic pots. Fresh leaves (0.25 g) were taken from each treatment and placed overnight chlorophyll extracted with 80% acetone at 0.4°C. These extractions were centrifuged at 10,000 rpm for 5 min. The supernatant obtained was used for measuring absorption pattern at wavelength of 663, 645 and 480 nm by using spectrophotometer (Hitachi-U2001, Tokyo, Japan).

### 2.6-Extraction of anti-oxidant enzymes

Antioxidant enzymes were extracted from the leaf collected samples. The leaf tissues (ca. 0.5g) were grounded by mortar and pestle in 5mL of 50mM using chilling phosphate buffer. The mixture was filtered and centrifuged at 15000 rpm for 20 minutes at 4⸰C. Activity potential of antioxidant enzymes were studied as follow using the mixture prepared.

#### 2.6.1-Estimation of superoxide dismutase (SOD) activity

Super oxidase (SOD) functioning was measured by method of Giannopolitis and Ries [28]. The process depends upon principle of photochemical reduction inhibition of nitroblue tetrazolium (NBT) at 560 nm. SOD functioning was noted having reaction mixture of enzyme extract 50 uL used in solution with 50 uM NBT, 1.3 uM Vitamin B2 (riboflavin), 13 mM methionine, 75 nMEDTA, 50 mM phosphate buffer (pH 7.8). Light source used for reacting solution of 30 W florescent in a chamber. When lamp turned on reaction started for 15 min and after turned it off, the reaction was ceased. The blue formazone which formed on photo reduction of NBT was calculated at 560 nm by using UV-visible spectrophotometer.

#### 2.6.2-Determination of Peroxidase (POD) activity

Method of Chance and Maehly [29] was used to determine the POD functioning. In reaction mixture POD is fully dependent on guaiacol oxidation. The reaction mixture contained 50mM phosphate buffer, 20 mM guaiacol, 40mM H_2_O_2_ and 100 uL enzymes extract. When guaiacol poured to solution reaction begins. Variations in absorbance patterns were noted after 20 sec, at 470 nm.

#### 2.6.3-Determination of Catalase (CAT) activity

The reaction solution made for CAT comprised of 50mM phosphate buffer (pH 7.8), 59 mM H_2_O_2_, and 0.1 mL enzyme extract following Chance and Meheley [29]. The decrease in the absorbance of the mixture was taken as disappearance of H_2_O_2_ as a basic phenomenon behind the estimation of CAT activity.

### 2.7-Malondialdehyde contents

The membrane lipid peroxidation was estimated by measuring quantity of malondialdehyde in the tissue described by Cakmak and Horst [30] with modest modifications. One gram of fresh leaf material was ground in 10 mL of TCA (10% solution prepared in dH_2_O). The supernatant (0.5 mL) as obtained from the homogenized material was mixed with 2 mL of 0.5% thiobarbituric acid (TBA), prepared in 20% TCA. Test tubes having the triturate were kept at 95°C for 50 min, and then cooled immediately in chilled water. After centrifugation (10,000× *g*) of mixture for 10 min, the absorbance of coloured part was read at 600 and 532 nm. The content of MDA was calculated using the formula:

MDA(nmol)=Δ(A532nm‐A600nm)/1.56×105

Absorption coefficient for the calculation of MDA is 156 mmol−1 cm−1.

### 2.8-Hydrogen peroxide contents

H_2_O_2_ contents were measured by using the method of Velikova *et al*., [31] with some minor modifications as required. Test mixture was prepared by homogenizing the fresh leaf (0.1g) with 5 mL volume of 0.1% TCA on an ice bath. The extract was centrifuged at 12000 rpm for 5 minutes. About 0.5 mL of test extract and 0.5 mL of Potassium Phosphate Buffer was taken in a test tube for further analysis. To reaction mixture 1mL of 1 M Potassium Iodide was incorporated. Then the mixture was shaken well before taking the reading at 390 nm using spectrophotometer.

### 2.9-Estimation of proline values

The method devised by Bates *et al*., [32]. was followed for the estimation of proline. Briefly, 0.1 g of leaf (fresh material) was crushed in 5 mL of sulfosalicylic acid (3%). After filtration, 100 μL of the extract was mixed well with 20 mL of 6 M phosphoric acid (2 mL each). Then, the mixture was reacted with glacial acetic acid (2 mL of each) and acidic ninhydrin heated the mixture in a water bath for 1 h at 95°C. After cooling well, 1 mL of toluene was mixed with reaction mixture and the optical density of colored phase was read at 520 nm. Proline concentration was measured following the equation:

Prolineμmolg−1Fw=mLoftoluene/115g×μgprolinemL−1)/sample(g)

### 2.10-Agronomic profile

#### 2.10.1-Estimation of seed starch contents

Starch contents were analyzed via iodine test in randomly selected rice caryopsis with glucose as a standard in accordance with the procedure given by Sullivan [33]. Each sample to be evaluated was mixed with 1 mL of iodine solution (4 g of potassium iodide and 1.27 g of iodine) for 10 min. Absorbance was measured at 660 nm with a spectrophotometer.

#### 2.10.2-Estimation of seed protein contents

Rice caryopsis were crushed into powder to analyse protein contents. Paddy protein contents were studied by using the method given by Gornall *et al*., [34]. In this method, bovine serum albumin was taken as the standard protein. Burette reagent was prepared by mixing 0.3 g of CuSO_4_·5H_2_O, 0.5 g of KI, and 0.9 g of sodium potassium tartrate in up to 100 mL of distilled water. The same concentration of reagent was mixed with standards as well as rice caryopsis and subjected to spectrophotometric analysis at 540 nm in accordance with the procedure.

#### 2.10.3-Yield profile

Current study evaluated yield profile of rice plants by counting number of tillers per plant, number of panicles per plant, number of spikelet per panicle, panicle length (cm), straw yield of rice (g) per pot, paddy yield of rice (g) per pot and 1000 paddy weight (g). A meter rod or measuring scale was used to determine the panicle length and a manual electronic balance was used to determine weight profile following previous protocol [25].

### 2.11-Statistical analysis

The data collected was incorporated on Microsoft excel sheet. For principal component analysis (PCA) and Spearman correlation software matrix XLSTAT version 2014 was used. The analysis of variance studies (ANOVA) and LSD test were performed using Co-STAT version 6.3 (developed by Cohort Software Berkley, CA, USA).

## 3-Results

### 3.1-Yield profile

**Fig 1:**
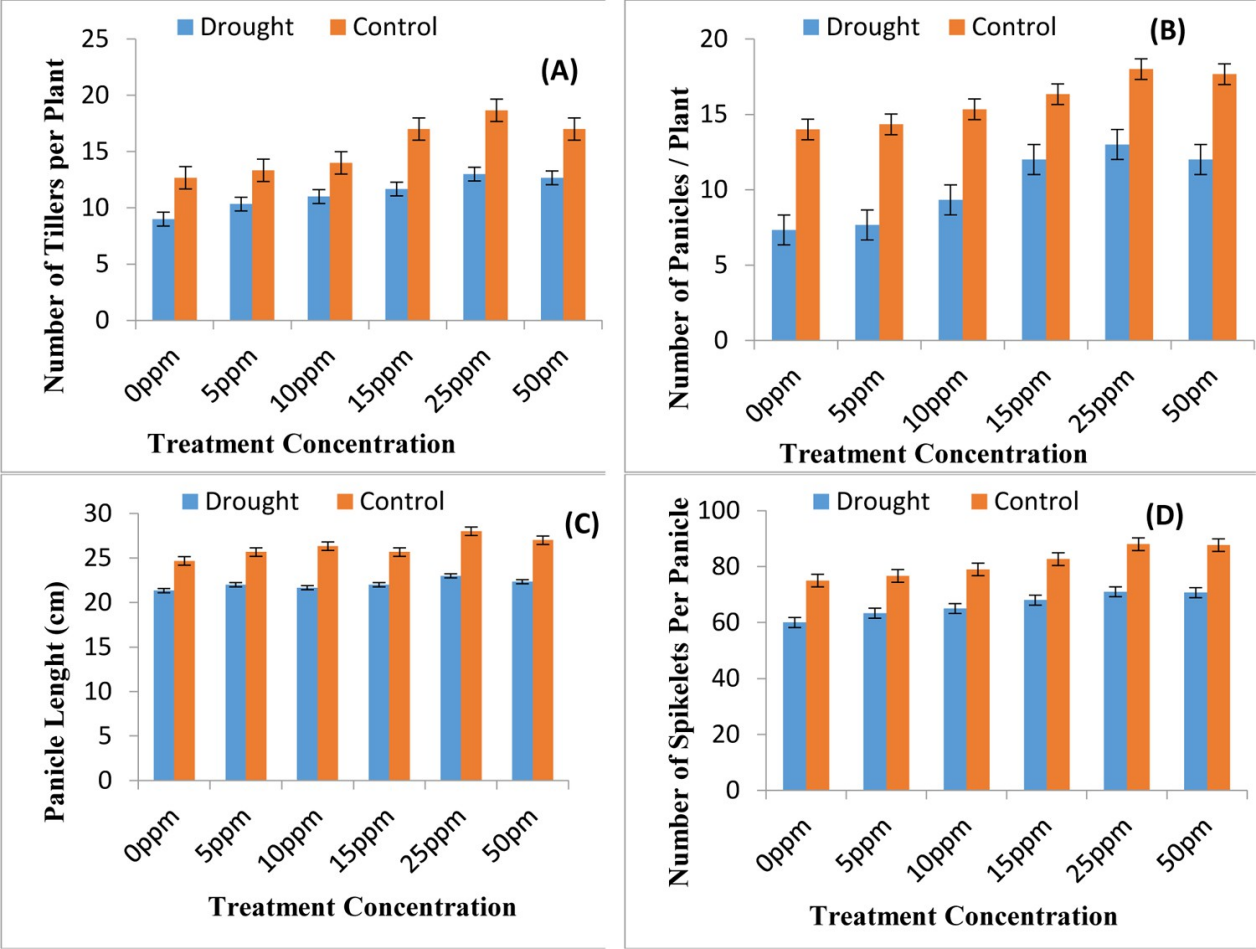
Yield attributes of rice plants. (A) Number of tillers per plant (B) Number of panicles per plant (C) Panicle length and (D) Number of spikelet per panicle as affected by various regimes of ZnONPs under water scarce and well irrigated environment (mean ± SE; n = 3).

Experimental results of the current study highlight the drought induced damages in yield quantity and quality of rice plants and subsequent improvement by ZnONPs priming treatments. The number of tillers of the rice plants under study were determined and experimental data has been given in Fig 1. The tiller count was directly linked to paddy yield and biomass production and it was explored that drought stress had decreased yield viz number of tillers of rice plants (Fig 1A). The data clearly shows that ZnONPs priming treatments applied at concentration 15ppm and 25 ppm which significantly increased the number of tillers of the rice plants and are helpful in alleviating the drought compromised yield.

**Fig 2:**
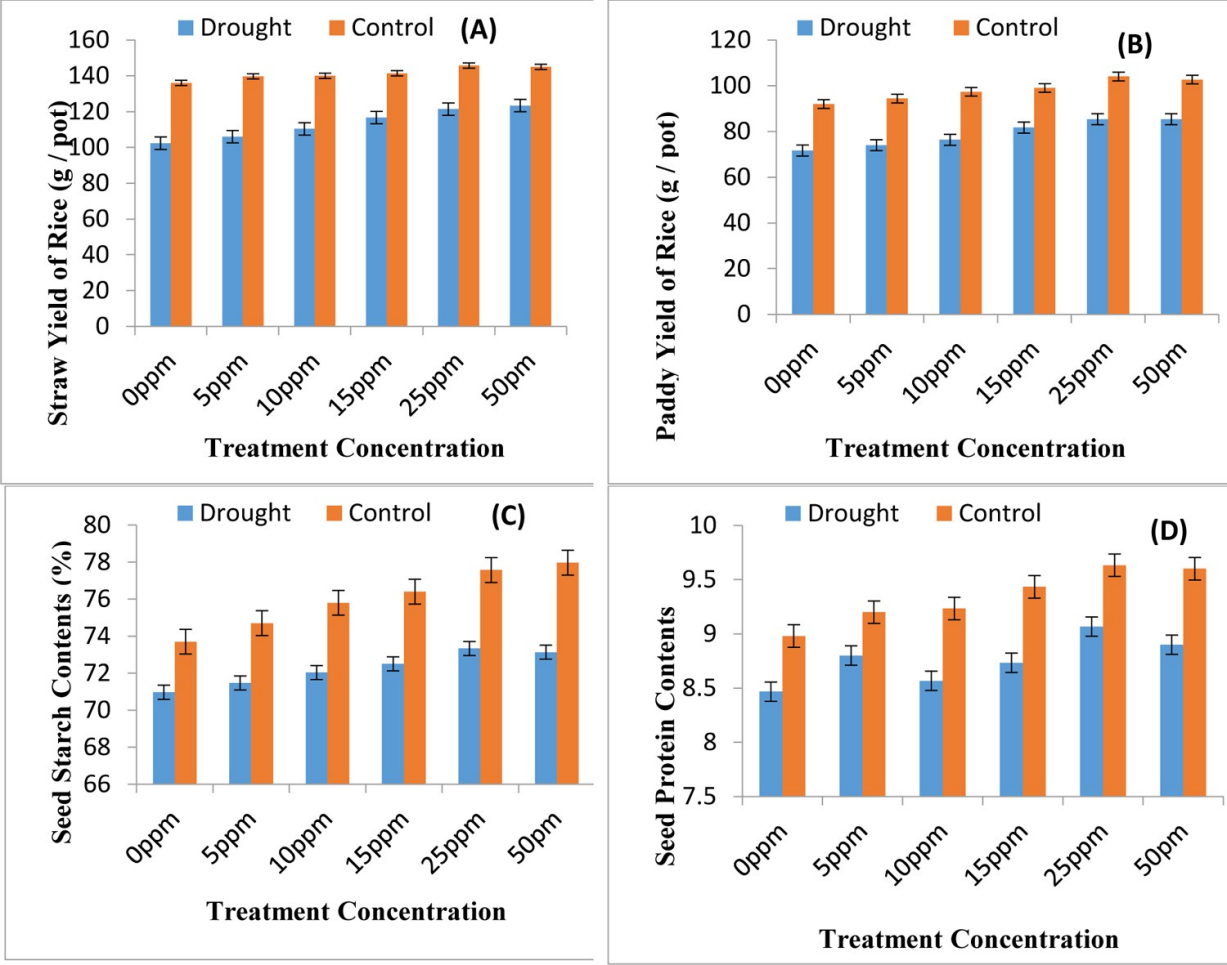
Yield attributes of rice plants. (A) Straw yield of rice (B) paddy yield of rice (C) seed starch contents and (D) seed protein contents as affected by various regimes of ZnONPs under water scarce and well irrigated environment (mean ± SE; n = 3).

The yield parameter related to panicle count was tested and data obtained has been presented in Fig 1B. Similarly the data related to panicle length and number of spikelet per panicle were also recorded in current study and has been presented in Fig 1C and 1D, respectively. The number of panicles per plant, panicle length and spikelet count per panicles are helpful in determining the rice caryopsis yield. The mentioned figures clearly indicated that water shortage environment leads to significant reduction in panicle count, panicle length and spikelet count per panicle leading to losses in yields. The seed priming with ZnONPs as pre sowing treatment has been found encouraging in removing drought induced reduction in yield attributes. All the concentrations of ZnONPs have been found encouraging however the 25ppm ZnONPs concentration has been found the best among all. Data presented in Fig 2 shows that water shortage environment leads to decrease in yield of rice in terms of both straw yield of rice and paddy yield of rice. The seed priming with ZnONPs is helpful in updating the yield profile of rice under both well irrigated and water deficit environment.

The mean value of paddy yield of rice and straw yield of rice per pot under water deficit environment without seed priming treatment is 71.6 and 102.3 grams, respectively and under well irrigated environment mean values were at 92 and 136 grams, respectively. The seed priming with 50 ppm ZnONPs increases these mean values by 85.333 and 123.333 grams, respectively under water deficit environment. Under well irrigated conditions the mean values of paddy yield per pot and straw yield per pot also increased significantly showing enhancement in their mean values by 104 and 145.77 grams, respectively (Fig 2A and 2B; Table 2).

**Table 2:** Mean square and p values from the ANOVA of data obtained through ZnONPs application on rice seeds.

Variation Source	*df*	NOT/P	NOP/P	PL	NSPP	H2O2	MDA
**Water Stress (WS)**	1	156.25*** (0.0000)	294.694*** (0.0000)	156.25*** (0.0000)	2070.25*** (0.0000)	342.25*** (0.0000)	121 *** (0.0000)
**Priming Treatment (PT)**	5	22.4277*** (0.0000)	24.9833 *** (0.0000)	4.3611 ns (0.0625)	145.11 *** (0.0000)	86.2944*** (0.0000)	8.4891*** (0.0000)
**WS X PT**	5	1.98333*** (0.0000)	1.29444 ns (0.0625)	0.716 *** (0.0000)	3.5166 ns (0.0562)	0.65 ns (0.8957)	5.6186*** (0.0000)
**Error**	24	0.91666	0.5257	1.7777	1.38388	2.0277778	0.0980
**Variation Source**	***df***	**SYR**	**PYR**	**SS**	**SP**	**PRO**	**CAT**
**Water Stress (WS)**	1	7028.027*** (0.0000)	3306.25*** (0.0000)	128.822*** (0.0000)	3.14471*** (0.0000)	604.053*** (0.0000)	853.61*** (0.0000)
**Priming Treatment (PT)**	5	214.9611*** (0.0000)	165.0277*** (0.0000)	9.87627*** (0.0000)	0.3153*** (0.0003)	15.2780* (0.109)	57.0416*** (0.0000)
**WS X PT**	5	39.4944*** (0.0000)	3.91666 * (0.02333)	0.8171 * (0.0296)	0.0217ns (0.7660)	4.23963 ns (0.4058)	5.0442 *** (0.0001)
**Error**	24	2.88888	2.47222	0.27027	0.042711	3.9933	0.5608
**Variation Source**	***df***	**1000 Wt**	**PH**	**PFW**	**PDW**	**SOD**	**POD**
**Water Stress (WS)**	1	195.533*** (0.0000)	633.445*** (0.0000)	378.9511*** (0.0000)	18.2044*** (0.0000)	1536.104*** (0.0000)	742.835*** (0.0000)
**Priming Treatment (PT)**	5	7.28512*** (0.0000)	67.3790*** (0.0000)	11.35311*** (0.0000)	0.2913ns (0.1666)	50.1441*** (0.0000)	31.337*** (0.0000)
**WS X PT**	5	1.37627* (0.0235)	1.2032 ns (0.1376)	0.9137 ns (0.0954)	0.3704 ns (0.0881)	1.9610*** (0.0000)	1.5954 ns (0.8300)
**Error**	24	0.42944	0.6441694	0.4275	0.16861	0.87445	0.71202

ns non-significant, df. degree of freedom, NOT/P Number of Tiller per Plant, NOP/P Number of panicles per plant, PL Panicle length, NSPP Number of Spikelet per Panicle, H2O2 Hydrogen peroxide, MDA Malandialdehyde, SYR Straw Yield of Rice, PYR Paddy Yield of Rice, SS Seed Starch, SP Seed Protein, PRO Proline, CAT Catalase, 1000 Wt. 1000 Paddy Weights, PH Plant Height, PFW Plant Fresh Weight, PDW Plant Dry Weight, SOD Superoxide Dismutase, POD Peroxidase.

*, ** and *** = significant at 0.05, 0.01, and 0.001 levels, respectively.

The data presented in Fig 2C and 2D describes experimental results for seed vigour parameters seed starch and seed protein, respectively. Water shortage environment reduces the grain starch and protein values significantly leading to poor endospermic values. The nano priming with ZnONPs proves beneficial in raising seed starch and protein values overall seed vigour. The experimental data of 1000 paddy weight is being presented in Fig 4A, which clearly depicts that 1000 paddy weight of rice significantly decreases upon exposure to water shortage conditions. Various nano priming treatment concentrations of ZnONPs were used as drought ameliorating agents. All the treatments affected the 1000 paddy weight differentially however priming with ZnONPs used at concentration of 25ppm has been found more suitable for increasing the 1000 paddy weight of rice plants under both well irrigated and water shortage conditions.

### 3.2-Total chlorophyll and levels of osmotic stress indicators

**Fig 3:**
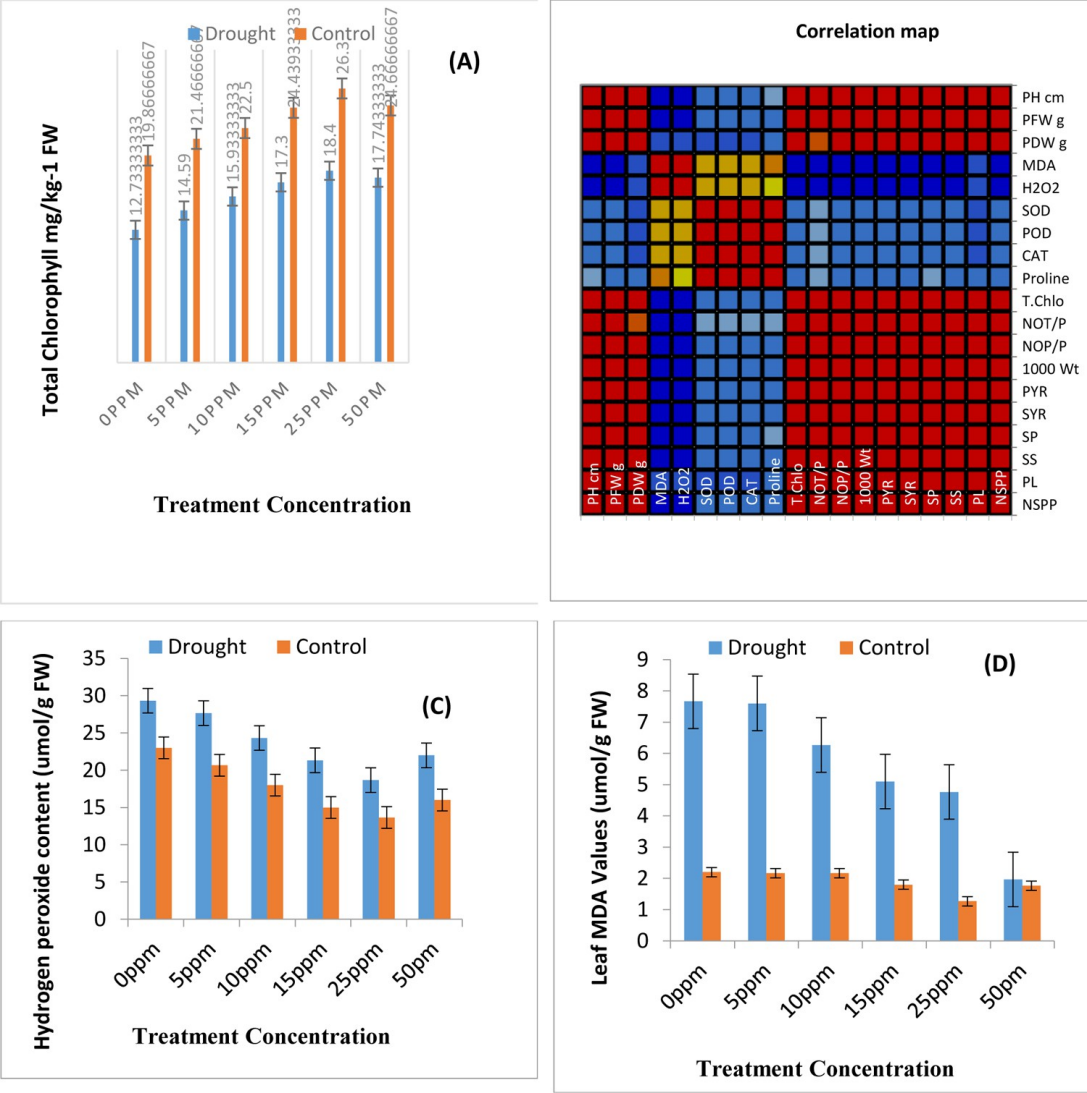
A. Total chlorophyll values of plants under current study. B. Spearman Correlation map of all studied attributes. Values of some biochemical attributes and antioxidant enzymes activities in rice plants (C) H_2_O_2_ (D) Malondialdehyde contents.

To improve the yield profile a plant must produce photosynthetic pigments efficiently. Fig 3A represents the total chlorophyll contents of rice plants which is associated with yield of seed. The bar chart shows significant decline in values of total chlorophyll upon imposition of drought stress. The seed priming with ZnONPs has been fruitful in enhancing production of total chlorophyll contents. A look at Fig 3B provides the idea that there exists strong correlation between increase in total chlorophyll and yield attributes. Data presented in Fig 3C and 3D indicates elevated levels of osmotic stress indicators Hydrogen peroxide and Malondialdehyde contents, respectively upon exposure of experimental rice pots to water deficit environment. Water stress leads to damages in lipid bilayer structure of biological membranes and as a result MDA accumulation takes place. Seed priming treatments leads to significant reduction in MDA contents of rice plants both in control and drought stress conditions. Seed priming with ZnONPs results in 53% reduction in MDA contents of water stressed rice plants. The values of ANOVA mentioned in Table 2 showed that ZnONPs are highly significant in decreasing MDA contents.

Water stress leads to increased accumulation of hydrogen peroxide as shown in Fig 3C. Nano priming with ZnONPs mitigates hydrogen peroxide levels by decreasing them indicating lowering of osmotic stress. All the treatments affect hydrogen peroxide level in both water stress and controlled environment however the decreasing effect of a treatment on the parameter is treatment specific.

### 3.3-Biomass production

**Fig 4:**
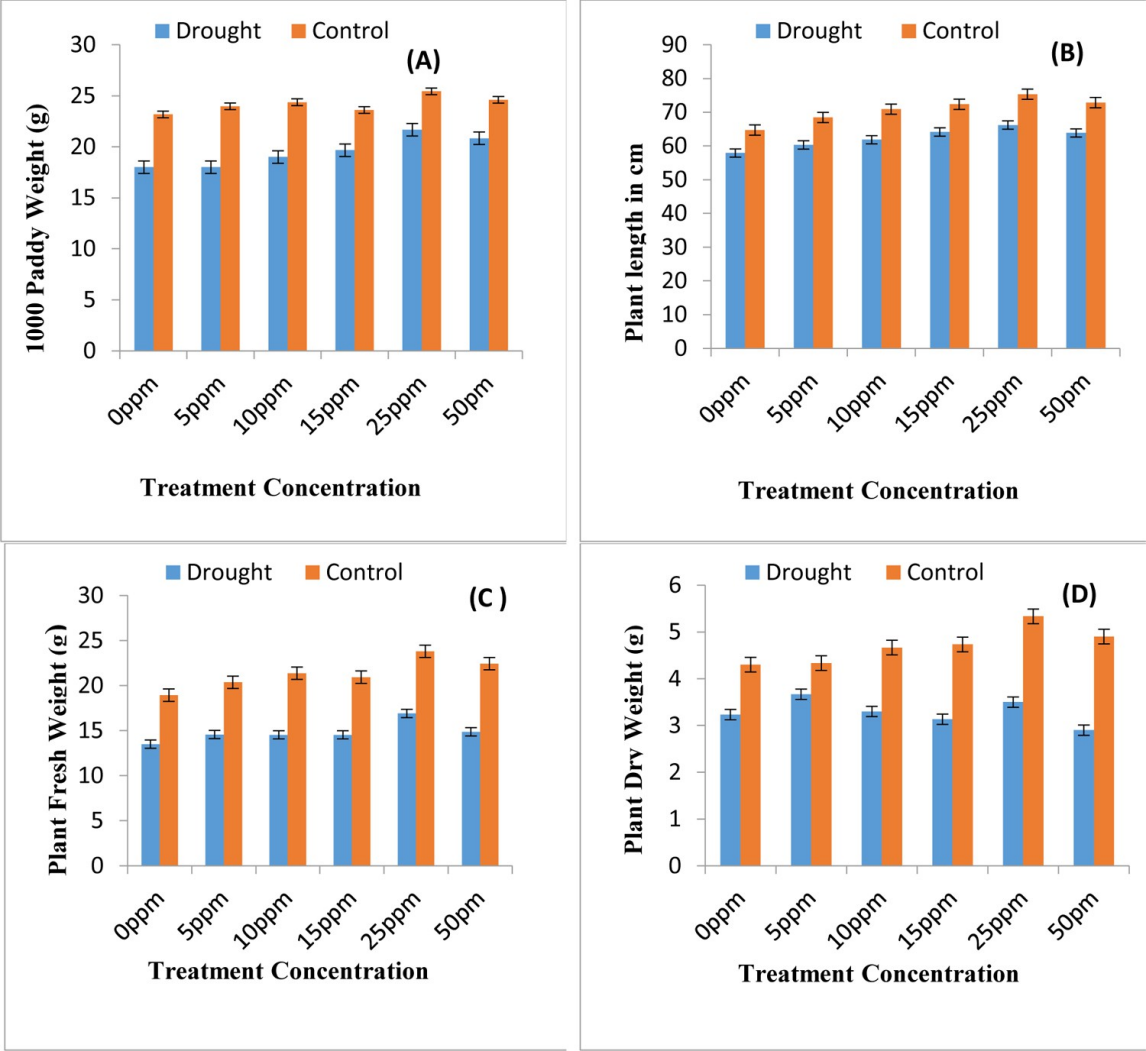
Yield and growth attributes of rice plants. (A) 1000 paddy weight (B) height of plants (C) Plant fresh weight and (D) plant dry weight as affected by various regimes of ZnONPs under water scarce and well irrigated environment (mean ± SE; n = 3).

**Fig 5:**
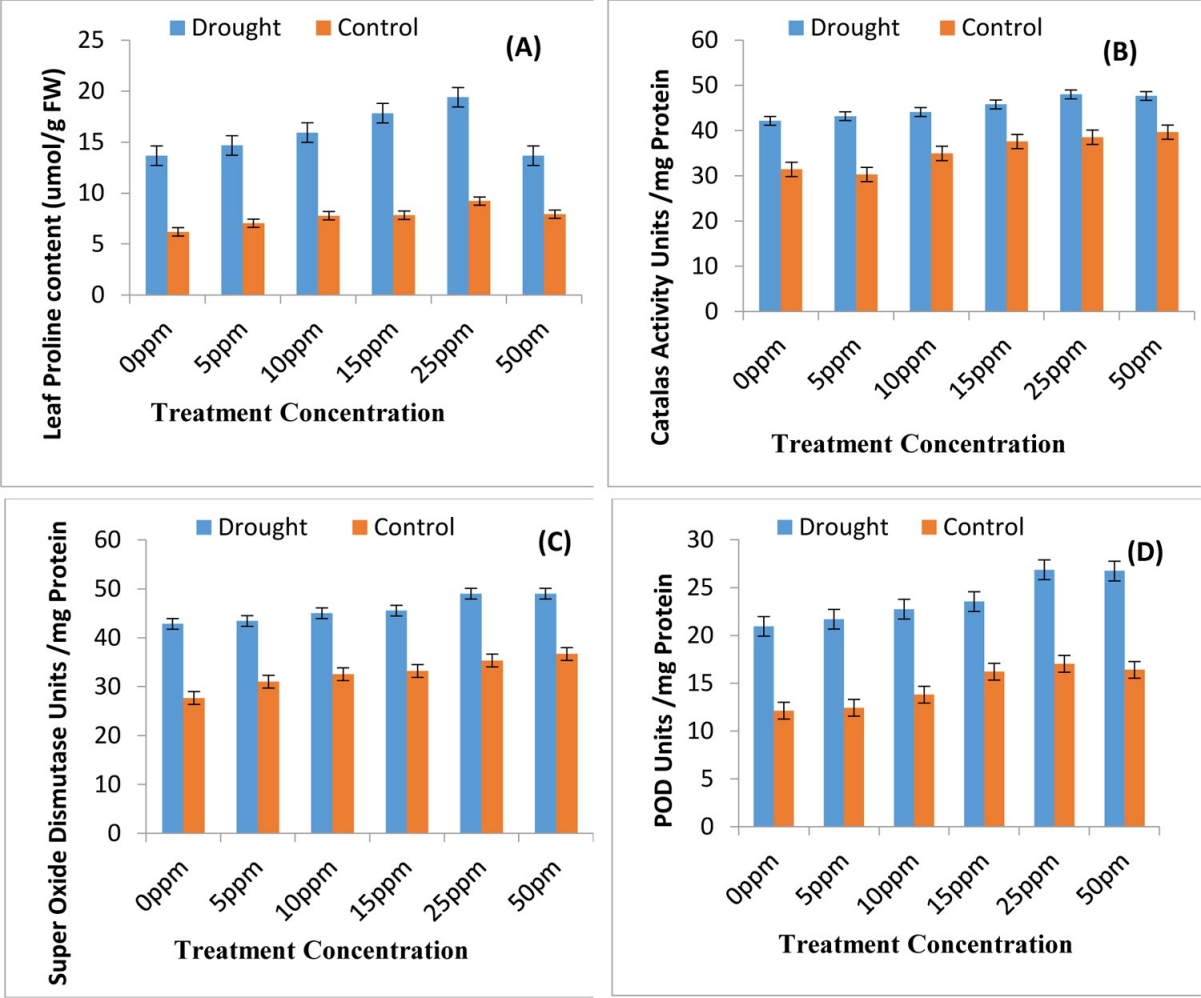
Values of some biochemical attributes and antioxidant enzymes activities in rice plants. (A) Proline values (B) Catalase (C) Super Oxide Dismutase (D) Peroxidase activities as affected by various regimes of ZnONPs under water scarce and well irrigated environment (mean ± SE; n = 3).

The study revealed that increase in total chlorophyll values also lead to increase in biomass production. Data regarding growth attributes plant height, plant fresh weight and plant dry weight has been presented in Fig 4B–4D, respectively. Drought stress leads to reduction in plant height by 31%, plant fresh weight by 22% and plant dry weight by 28%. Seed priming treatments imparted in current study show significance increase in plant biomass. There is highly significant correlation of plant height with plant fresh weight and plant dry weight (0.9267*** and 0.8245***, respectively). Data presented in Fig 5A represents increased proline levels under the water stress conditions. Priming with ZnONPs further enhances the levels of proline amino acid facilitating the plant to combat water shortage stress.

### 3.4-Analysis of activities of anti-oxidant enzymes

The activities of all the anti-oxidant enzymes are increased in rice plants upon induction of water stress (Fig 5A–5D). The drought stress leads to increase in activities of SOD, CAT and POD by 67%, 33% and 92%, respectively. A further elevation in activities of SOD, CAT and POD takes place in rice plants raised from ZnONPs primed seeds by 11%, 13% and 38%, respectively. The best effects in increasing the modulation of anti-oxidant enzymatic machinery takes place from ZnONPs used at 25 ppm concentration however the 50 ppm concentration is also fruitful in enhancing the potentials of anti-oxidant enzymes.

## 4. Discussion

Drought stress significantly affects the performance of crops in terms of their nutrient profile, growth and agronomic vigour [2]. Nano seed-priming is an efficient tool to combat climate change induced drought and other abiotic stresses [35]. Current study presents analysis of variance studies of the data obtained from rice plants raised through nano ZnO primed seeds. Present data obtained through analysis of total chlorophyll contents revealed that total chlorophyll contents were decreased significantly upon exposure to drought stress. The rice plants raised from ZnONPs primed seeds enhanced the plant biomass which is indicator of active photosynthetic machinery. Similar results have been reported by Rizwan *et al*., [36] on wheat plants. The increase in chlorophyll contents may promote carboxylation and enzymatic machinery of C3 plants [37].

The enhanced activities of antioxidant enzymes (CAT, SOD and POD) under drought were examined in current study. This enhancement in enzymatic activities is a part of plant internal defence mechanism to combat abiotic stresses [7]. The seed priming treatments with ZnONPs further enhanced the activities of these antioxidant enzymes proving their efficacy in drought amelioration. These results are in accordance with previous study reported by Itroutwar et al. [38], where the efficacy of biogenic ZnONPs has been documented in rice plants. The enhanced SOD activity might be due to Zn acting as activator for the enzyme [39]. Increased activities of POD cause decomposition of hydrogen peroxide into water and oxygen [36].

The values of hydrogen peroxide and malondialdehyde (MDA) were also elevated in rice plants upon imposition of water shortage conditions. The elevated levels of these osmotic stress indicators were mitigated effectively by ZnONPs treatment. The decrease in lipid peroxidation product MDA might be due to nanoparticles mediated membrane recovery [40]. Similar behaviour of Zn nanoparticles primed Lupin seeds has been reported by Latef et al., [41].

Water shortage leads to decrease in plant height, plant fresh weight and dry weight in rice plants. The seed priming with ZnONPs improves the growth and morphology of rice plants (Table 3). These results are in compliance with a study conducted by Khan *et al*., [26], where similar results have been reported while experimenting with AgNPs primed pearl millet seeds. The increase in plant height might be due to Zn acting as activator in biosynthesis of amino acid tryptophan which is involved in the biosynthetic pathways of auxin [36].

**Table 3:** Spearman correlation matrix for studied variables and yield attributes of rice plants raised from ZnONPs primed seeds.

Variables	NOT/P	NOP/P	1000 Wt	PYR	SYR	SP	SS	PL	NSPP
**PH cm**	0.9501***	0.9648***	0.9338***	0.9709***	0.9546***	0.9131***	0.9746***	0.8553***	0.9709***
**PFW g**	0.8833***	0.9112***	0.9074***	0.9424***	0.9306***	0.8525***	0.9368***	0.8919***	0.9360***
**PDW g**	0.7767***	0.8328***	0.8431***	0.8575***	0.8309***	0.8289***	0.8487***	0.8861***	0.8478***
**MDA**	-0.8927***	-0.8980***	-0.8646***	-0.9214***	-0.9282***	-0.8054***	-0.9148***	-0.7532***	-0.9100***
**H2O2**	-0.9168***	-0.9050***	-0.8452***	-0.8916***	-0.8800***	-0.8818***	-0.9007***	-0.7614***	-0.9005***
**SOD**	-0.3638***	-0.5174***	-0.5514***	-0.5201***	-0.5195***	-0.4399***	-0.4940***	-0.6083***	-0.5221***
**POD**	-0.3467***	-0.5168***	-0.5472***	-0.5241***	-0.5336***	-0.4209***	-0.5032***	-0.6356***	-0.5222***
**CAT**	-0.3647***	-0.5102***	-0.5607***	-0.5177***	-0.5327***	-0.4448***	-0.4983***	-0.6111***	-0.5184***
**Proline**	-0.3422***	-0.4694***	-0.5136***	-0.5161***	-0.5157***	-0.3457***	-0.5064***	-0.5241***	-0.4842***
**T.Chlo**	0.9310***	0.9717***	0.9480***	0.9833***	0.9717***	0.8794***	0.9788***	0.8590***	0.9709***
**NOT/P**	1	0.9234***	0.9005***	0.9462***	0.9364***	0.9082***	0.9461***	0.8277***	0.9452***
**NOP/P**	0.9234***	1	0.9507***	0.9778***	0.9714***	0.9173***	0.9788***	0.8648***	0.9819***
**1000 Wt**	0.9005***	0.9507***	1	0.9611***	0.9499***	0.8878***	0.9552***	0.8790***	0.9507***
**PYR**	0.9462***	0.9778***	0.9611***	1	0.9828***	0.9134***	0.9907***	0.8965***	0.9926***
**SYR**	0.9364***	0.9714***	0.9499***	0.9828***	1	0.9003***	0.9801***	0.8829***	0.9851***
**SP**	0.9082***	0.9173***	0.8878***	0.9134***	0.9003***	1	0.9177***	0.8620***	0.9245***
**SS**	0.9461***	0.9788***	0.9552***	0.9907***	0.9801***	0.9177***	1	0.8830***	0.9842***
**PL**	0.8277***	0.8648***	0.8790***	0.8965***	0.8829***	0.8620***	0.8830***	1	0.8977***
**NSPP**	0.9452***	0.9819***	0.9507***	0.9926***	0.9851***	0.9245***	0.9842***	0.8977***	1

Values with *** are different from 0 with a significance level alpha = 0.05.

NOT/P Number of Tiller per Plant, NOP/P Number of panicles per plant, PL Panicle length, NSPP Number of Spikelet per Panicle, H2O2 Hydrogen peroxide, MDA Malandialdehyde, SYR Straw Yield of Rice, PYR Paddy Yield of Rice, SS Seed Starch, SP Seed Protein, PRO Proliine, CAT Catalase, 1000 Wt. 1000 Paddy Weights, PH Plant Height, PFW Plant Fresh Weight, PDW Plant Dry Weight, SOD Superoxide Dismutase, POD Peroxidase.

Various researchers have documented increase in plant biomass upon treating seeds with nanoparticles priming [26, 36, 42]. Seed priming strategies might be helpful in transforming crops into climate change resilient crops [43].

The agronomic profile of a plant is indicator of its proper homeostasis and physiological wellbeing culminating into good yield. In present study, starch and protein contents of rice caryopsis were examined and found depressed upon exposure to osmotic stress. The ZnONPs based seed pre-conditioning improved the starch and protein contents of the paddies. Data presented in the table shows spearman correlation matrix. The data presented shows that all of the studied variables have strong correlation with yield attributes of rice plants. Increase in agronomic and growth attributes might be due to ZnONPs mediated enhancement in amylase activities resulting in nutrient uptake and mobilisation [43]. To make agronomic profile of the rice plants the parameters such as number of tillers per plant, number of panicles per plant, number of spikelet per panicle, panicle length, straw yield of rice per pot, paddy yield per pot and 1000 paddy weight was recorded. All of the studied agronomic attributes were found declined upon imposition of drought stress. The decrease in yield characteristics is reflection of poor nutrient acquisition patterns due to water stress [44]. The seed priming with ZnONPs increases all the yield attributes and the optimum effects were observed with 25ppm concentration. The improvement in agronomic profile of maize plants might be due to increased rates of photosynthesis induced by priming treatments. Similar results of priming treatments with ZnONPs in maize have been reported by Tondey *et al*., [45]. The potential of biogenic zinc nanoparticles priming in rice has been documented by Itroutwar *et al*., [38] proving better biofortification of rice plants by seed priming measures. Furthermore experiments of Yasmeen *et al*., [46] reported that nano-priming with copper and iron nanoparticles in wheat is fruitful in increasing spike length, number of grains per spike and grain weights. The increase in nutrient profile of rice by nano-priming with Zinc oxide nanoparticles might be due to increased biosynthesis of enzymes involved in nutrient uptake and acquisition [47].

**Fig 6:**
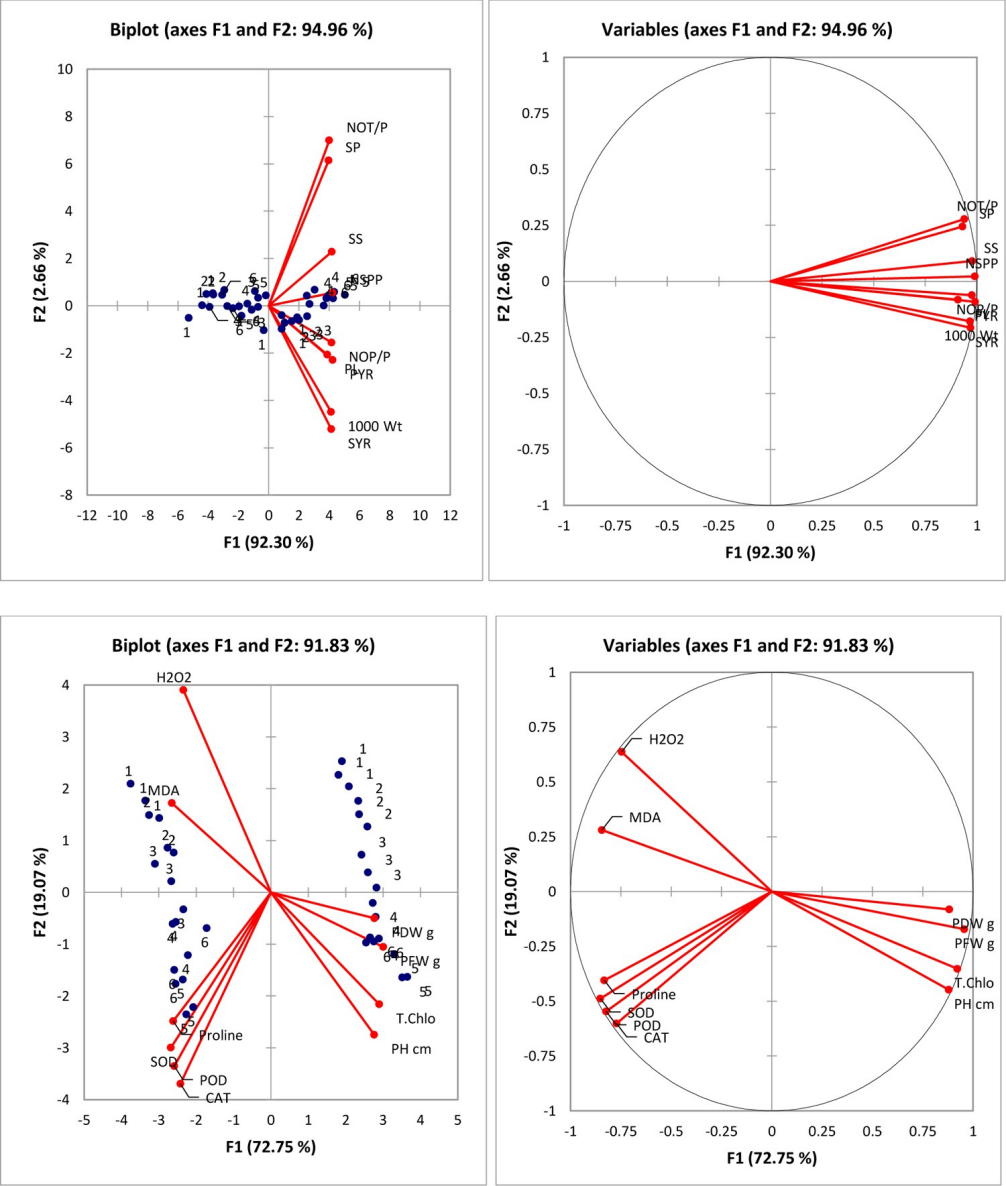
PCA analysis showing correlations among studied parameters of water-stressed rice plants primed with ZnONPs. NOT/P Number of Tiller per Plant, NOP/P Number of panicles per plant, PL Panicle length, NSPP Number of Spikelet per Panicle, H2O2 Hydrogen peroxide, MDA Malandialdehyde, SYR Straw Yield of Rice, PYR Paddy Yield of Rice, SS Seed Starch, SP Seed Protein, PRO Proline, CAT Catalase, 1000 Wt. 1000 Paddy Weights, PH Plant Height, PFW Plant Fresh Weight, PDW Plant Dry Weight, SOD Superoxide Dismutase, POD Peroxidase.

The principal component analysis (PCA) results of current study showed a strong association for growth and physio-biochemical attributes of rice plants along with agronomic parameters with ZnO priming treatments. These results are in accordance with the previous study of Tondey *et al*., [48–50] where similar association has been documented in case of maize plants [Fig 6].

Similarly, the results documented in this study depicted a strong correlation and association to priming treatments as deciphered by increased grain weight per plant, increased plant height and spike length where these outcomes are coincidence with previous work performed by Popovi´c *et al*., [48, 51, 52]. The drought stress or any biotic stress such pathogenic attack on crops has also drastic impact on yield of crops and it was reported in study of Capsicum crop [53]. The other cereal crops which are under stress have been explored for heat stress in rice and the comprehensive review was presented by the researchers [54, 55]. This plethora of rice crop has been addressed by mitigating mechanism of heat stress similarly zin oxide priming may also be used improvement of yield. The nano technology can also be useful not only in field of crops but also for production of biodiesel from seeds of plants and lot of work has been conducted on this field [56]. This use of nano technology through use of seed priming in terms of ZnOxidees and which be assisting in enhancing the yield of seed or grain of the crops as well and it will boost agronomic yield of the country.

## 5. Conclusion

Nano priming with ZnONPs of rice seeds is a promising field for exploitation in agricultural industry as deciphered by the results presented in this research paper. Keeping in view climate change mediated food insecurity such applications might prove beneficial in future. Seed priming with ZnONPs is helpful in water stress mitigation by modulating anti-oxidant enzymes and osmolytes accumulation. ZnONPs are potential candidates in updating the agronomic profile of rice by increasing yield traits. Current study recommends the nano priming with ZnONPs for rice seeds as pre sowing treatment. However further experimentation in field is necessary and it is recommended to explore the potential of nano seed priming in other crops as well.
